# Infection by Adenovirus Type 2 in a Short-Tailed Bat in Mexico

**DOI:** 10.1155/crve/2431526

**Published:** 2025-02-24

**Authors:** Armando Trejo-Chávez, Uziel Castillo-Velázquez, Adriana Méndez-Bernal, Karina Flores-Martínez, Gustavo Hernández-Vidal, Luis E. Rodríguez-Tovar, José P. Villarreal-Villarreal

**Affiliations:** ^1^Cuerpo Académico de Patobiología, Facultad de Medicina Veterinaria y Zootecnia de la Universidad Autónoma de Nuevo León, Escobedo, Nuevo León, Mexico; ^2^Cuerpo Académico de Zoonosis y Enfermedades Emergentes, Facultad de Medicina Veterinaria y Zootecnia de la Universidad Autónoma de Nuevo León, Escobedo, Nuevo León, Mexico; ^3^Unidad de Microscopía Electrónica del Departamento de Patología, Facultad de Medicina Veterinaria y Zootecnia de la Universidad Nacional Autónoma de México, Ciudad de México, Mexico; ^4^Departamento de Patología, Facultad de Medicina Veterinaria y Zootecnia de la Universidad Autónoma de Nuevo León, Escobedo, Nuevo León, Mexico; ^5^Cuerpo Académico de Epidemiología, Facultad de Medicina Veterinaria y Zootecnia de la Universidad Autónoma de Nuevo León, Escobedo, Nuevo León, Mexico

**Keywords:** Adenovirus Type 2, *Carollia perspicillata*, Mexico

## Abstract

A short-tailed female bat (*Carollia perspicillata*), 1 year old, died without apparent signs of the disease while on display at an amusement park in the Municipality of Montemorelos, Nuevo León, Mexico. Amphophilic nuclear inclusion bodies were observed in the intestinal epithelia, corresponding to the virus of the adenovirus genera. Although there have been no reported adenovirus cases of this bat species in Mexico, through an anatomopathological study and support from the microscopic, ultrastructural, and molecular findings of intestinal lesions, a diagnosis of enteritis caused by Adenovirus Type 2 was made. To the authors' knowledge, the case described herein is the first report of infection by Adenovirus Type 2 in a short-tailed bat in Mexico.

## 1. Introduction

Bats are considered the most abundant vertebrate mammals and have the most variability of species worldwide, except Antarctica [1]. They have been recognized as animals that harbor various pathogens, including some viruses with capacities, such as rabies virus [2]. However, some bat-borne viruses are classified as producers of emerging diseases, such as the Nipah virus recently discovered in Malaysia, Singapore, and Bangladesh [3, 4]; the Marburg virus found in Kenya, Gabon, and Republic of Congo [5]; the coronavirus that causes severe acute respiratory syndrome, which was recently described in the People's Republic of China [6]; and viruses causing Ebola and Hendra, found in West Africa and Australia, respectively [7]. These viruses have crossed the species barrier, infecting humans and domestic and wild animals. Other viruses, such as those from the Parvoviridae, Circoviridae, Picornaviridae, Adenoviridae, Poxviridae, Astroviridae, and Coronaviridae families, have been recently discovered in guano samples by molecular techniques [8]. Recent studies of polymerase chain reaction (PCR) and virus isolation on bats from Japan, Germany, and China have reported a new virus called RV1 from the family Adenoviridae, in fruit bats, as in *Pteropus dasymallus yayeyamae*, and in insectivorous bats, namely, in the species *Pipistrellus pipistrellus*, *Myotis* sp., and *Scotophilus kuhlii*. This virus causes severe gastrointestinal problems in these animals, suggesting that its mode of transmission is by direct contact [9, 10]. In contrast, the presence of other adenoviruses in bats has been described, such as the one reported in straw-colored fruit bats (*Eidolon helvum*) in Zambia [11], great fruit-eating bat (*Artibeus lituratus*), and lilium fruit bat (*Sturnira lilium*) in Brazil [12] and the Rafinesque's big-eared bat (*Corynorhinus rafinesquii*) in the United States [13], in addition to some bat species of the Vespertilionidae and Rhinolophidae families in Spain [14]. The Adenoviridae family is characterized by a wide range of guests. Virions are described as nonenveloped, with icosahedral symmetry, 70–90 nm in diameter, with a genome consisting of a single lineal molecule of double-stranded DNA of 26–48 kbp in size, with inverted terminal repeats. According to the International Committee on Viral Taxonomy, the Adenoviridae family is composed of seven different genera: *Mastadenovirus*, which infects only mammals; *Aviadenovirus*, which affects birds; *Atadenovirus*, which infects a wide range of guests, including ruminants and poultry; *Ichtadenovirus*, such as the one from the white sturgeon (*Acipenser transmontanus*); and *Siadenovirus*, which includes one of the frog adenoviruses and Adenovirus 3 in turkeys, in addition to the recently described virus in birds of prey, parrots, and turtles. On the other hand, *Testadenovirus* and *Barthaadenoviru*s are also considered [15]. Due to their ability to host and spread these microorganisms over long distances, a classification was started in 2008 based on the first isolation made in Japan in a Ryukyu flying fox (*P. dasymallus yayeyamae*) known as BtAdV1-FBV1, later in Germany in a common pipistrelle (*Pipistrellus pipistrellus*) where a second strain known as BtAdV-2 PPV1 was identified, in addition to the first completely sequenced adenovirus genome of the TJM strain of BtAdV-3 from a Rickett's large-legged bat (*Myotis ricketti*) [14]. With data from different reports on adenoviruses found in these species, the International Committee on Viral Taxonomy renamed the TJM strain from BtAdV-3 and the BtAdV-2 PPV1 strain as Bat *Mastadenovirus* A and B, respectively [11, 16]. Currently, the International Committee on Taxonomy of Viruses has designated other mastadenoviruses with letters according to the bat species in which they were found, including Bat *Mastadenovirus* C–J [11, 13, 17–19].

## 2. Case Presentation

**Figure 1:**
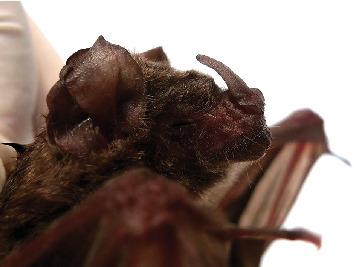
Specimen with fair body condition.

**Figure 2:**
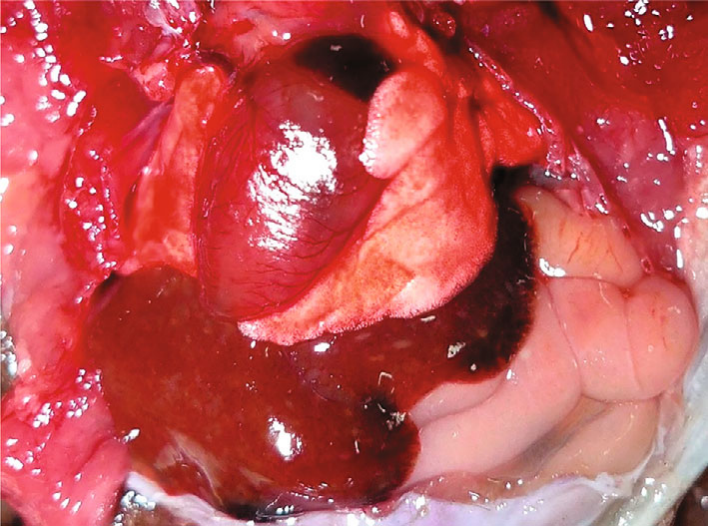
Marked intestinal distention with yellow semipasty material.

A female short-tailed bat (*Carollia perspicillata*), 1 year old, died suddenly while on display at an amusement park in the Municipality of Montemorelos, Nuevo León, Mexico. The animal was sent to the Pathology Department, Faculty of Veterinary Medicine and Zootechnics, Autonomous University of Nuevo León, for postmortem examination. At the external inspection, the bat showed regular body condition (Figure 1). The oral, palpebral, anal, and vaginal mucosae were extremely pale, showing anemia. The brain, lungs, and liver showed moderate and diffuse congestion at the gross examination. The lumen of the small and large intestines contained a yellowish semipasty material (Figure 2). The spleen and kidneys showed no apparent pathological changes.

**Figure 3:**
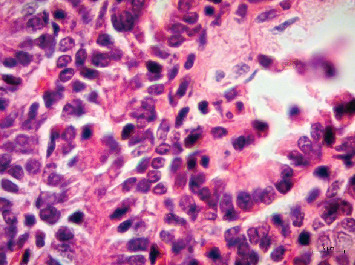
Presence of amphophilic nuclear inclusion body in intestinal epithelia. Hematoxylin and eosin. Bar = 5 *μ*m.

Samples of several organs were collected and fixed in 10% buffered formalin for routine histopathological processing. Microscopically, the lungs showed marked thickening of the alveolar septa and interstitial inflammatory infiltrate of the lymphocytic type. The epithelium of the trachea, bronchi, and bronchioles denoted hyperplasia. The villi of the small and large intestines had an inflammatory infiltrate of lymphocytes and plasma cells, as well as numerous amphophilic nuclear inclusion bodies (Figure 3). The white matter of the brain showed congestion and perivascular edema, microthrombosis, and discreet glial reaction.

**Figure 4:**
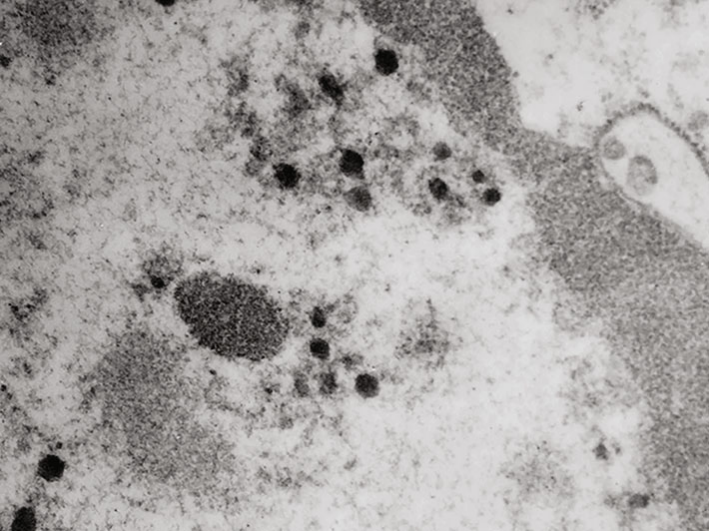
Numerous virions with icosahedral symmetry from 79 to 83 nm in diameter were observed in the nucleus of the enterocyte (arrows). Electrolucent vacuoles containing virions with a capsid of 63 nm in diameter, surrounded by an electron-dense layer of 150 nm in diameter, were observed near the nucleus (segmented arrow). TEM, 50,000×.

Transmission electron microscopy (TEM) was carried out on the formalin-fixed paraffin wax–embedded sample of the intestine. Briefly, the sample was dewaxed, rehydrated, and fixed in 2.5% glutaraldehyde. Afterwards, they were washed with cacodylate buffer and postfixed in 1% osmium tetroxide for 2 h. Again, the sample was washed with cacodylate buffer, dehydrated with increasing concentrations of acetone, and preincluded in a resin:acetone mixture for 72 h. Subsequently, it was included in epoxy resin for 24 h at 60°C. Semithin sections of 120 nm were made, which were stained with toluidine blue and evaluated in the optical microscope, in order to select the site of interest. Ultrathin sections of 60–80 nm were stained with uranyl acetate and lead citrate and examined in a Zeiss EM 900 TEM operated at 50 kV. Ultrastructurally, numerous virions with icosahedral symmetry, approximately 79–83 nm in diameter, were observed in the nucleus of enterocytes. In the regions close to the nucleus, electrolucent vacuoles containing virions with a capsid of 63 nm in diameter, surrounded by an electron-dense shell, which was 150 nm in diameter, were observed (Figure 4).

The PCR study was conducted from positive samples embedded in paraffin. For genomic DNA extraction, 20–40 paraffin sections of ≥ 2 mm (amounts to ~10–15 mg of deparaffinized tissue) were collected into a microcentrifuge tube. To remove paraffin, samples were incubated for 10 min at room temperature with 800 mL xylene and pelletized at 14,000 × *g*. This step was repeated if macroscopically visible paraffin remnants were present and the supernatant was still turbid. In most samples, one rinsing step was sufficient; some required two steps. Pellets were then resuspended and washed twice with 500 mL of 99% ethanol. After resuspending in 300 mL Cell Lysis Solution (Gentra Puregene Tissue Kit, Qiagen, Hilden, Germany) and thorough homogenization by pipetting up and down, the sample was heated to 65°C for 45 min, followed by 98°C for 15 min. After cooling and adding 1.5 mL proteinase K solution, the samples were incubated for 48 h at 56°C. During this time, 1.5 or 3.0 mL proteinase K solution was supplemented depending on the amount of tissue. RNA was then digested by adding 1.5 mL RNase A solution and incubating for 15 min at 37°C. The samples were placed on ice for 5 min after adding 100 mL of Protein Precipitation Solution (Gentra Puregene Tissue Kit) and centrifuged at 14,000 × *g* for 3 min. DNA in the supernatant was then precipitated by adding 300 mL of 100% isopropanol. The samples were incubated for 5 min on ice and centrifuged at 14,000 × *g* for 3 min. DNA pellets were washed twice in 300 mL of 70% ethanol, air-dried for 10 min, and resuspended in 50 mL DNA Hydration Solution (Gentra Puregene Tissue Kit). DNA concentration was determined and adjusted to 100 ng/mL by adding 5 mM Tris buffer (pH 7.4) [20]. The specific primers designed for sequence detection were Adenovirus Type 2 (Accession No. FJ983127) and the housekeeping gene (gapdh) of *C. perspicillata* (Accession No. NC_022422.1), which was used as an internal control in IDT's PrimerQuest software developed by the Whitehead Institute for Biomedical Research. DNA extracted from formalin-fixed and paraffin-embedded tissues was used as the template for PCR. Virus identification was performed by PCR testing using the following primers: sense 5⁣′-CGGTCAACCTGCAGAGATAAA-3⁣′ and antisense 5⁣′-GGAGATCCTATGGGAGCAAATG-3⁣′, which amplify an expected 429 bp product for Adenovirus Type 2, and sense 5⁣′-CGAGATGTCAACTACGGATGAG-3⁣′ and antisense 5⁣′-TAGGAGGTCGGGTGAGAATAG-3⁣′, which amplify an expected 540 bp product for *C. perspicillata*. Each PCR consisted of 1× reaction solution enhancer (Life Technologies Corporation, Carlsbad, CA, United States), 1× buffer, 2 *μ*M MgSO4, 0.2 *μ*L deoxynucleotide triphosphates, 1 pM of each primer, 5 U Taq polymerase (Life Technologies), 2 ng DNA, and 50 *μ*L molecular biology grade H_2_O, to a 50 *μ*L final volume. The amplification protocol consisted of an initial denaturing cycle at 95°C for 5 min, followed by 30 cycles at 94°C for 30 s, 55°C for 30 s, 72°C for 30 s, and a final extension cycle at 72°C for 5 min. Visualization of the amplification products was performed by gel electrophoresis in 1% (*w*/*v*) agarose gel with 1× TBS and stained in 1% Gel-Red.

**Figure 5:**
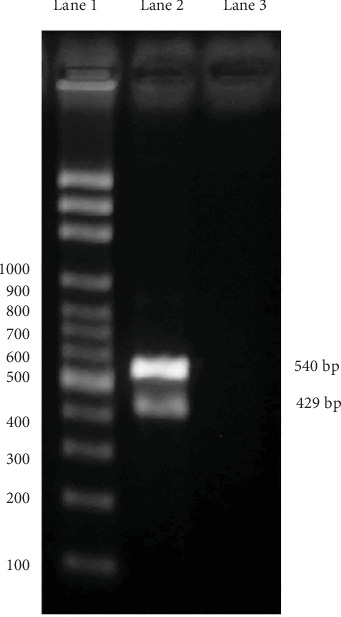
PCR amplification of Adenovirus Type 2 and *Carollia perspicillata*. Lane 1: molecular weight marker 100 pairs of bases. Lane 2: bat small intestine sample corresponding product of 540 bp of *C. perspicillata* and a product of 429 bp corresponding to Adenovirus Type 2. Lane 3: negative control (no DNA template).

At PCR testing, an expected 540 bp product for *C. perspicillata* and 429 bp product for Adenovirus Type 2 were made (Figure 5).

## 3. Discussion

The microscopic, ultrastructural, and molecular findings of the intestinal villus observed in this study suggested infection by Adenovirus Type 2. Virions observed in the digestive epithelia were consistent with previous reports of the new adenovirus experimentally studied in cultured kidney and spleen cells of the fruit bat (*P. dasymallus yayeyamae*) [9] and the purified supernatant obtained from Vero E6/7 cell cultures in this species [10]. To the authors' knowledge, this case is the first report of infection by Adenovirus Type 2 in a short-tailed bat in Mexico. The information provided by the amusement park showed that only this bat showed clinical signs and was the only specimen dead provided. Only several species of fruit bats are known to coexist and are on permanent display. The cause or source of the infection in this bat could not be determined in this study. However, the bat likely acquired the virus through direct contact with other persistently infected bats by sharing contaminated food or water or through aerosols [10]. However, the bat likely acquired the virus through direct contact with other persistently infected bats by sharing contaminated food or water or through aerosols [10]. In addition, the stress associated with handling the animal may have generated a state of immunosuppression, causing infection by this adenovirus and ultimately its death [21]. The virus can evade the immune response, remain viable in the bat for a time, and cause disease during an immunosuppressive event [21]. This could have happened in this case. It has been established that bats have important differences in the immune response to viral infection in relation to other mammals, such as rodents and primates [21]. This could be due to the large number and diversity of existing bat species. A low probability of cross-reaction between different pathogens of bat species has also been postulated due to the host range [16]. So far, adenoviruses found in this bat species have not caused infections in humans [10]. Therefore, early detection is essential for inappetent and depressed animals to separate and monitor them in case of death and conduct appropriate studies to determine the exact cause of death.

## Data Availability

The data that support the findings of this study are available from the corresponding author upon reasonable request.
